# Participative Spatial Scenario Analysis for Alpine Ecosystems

**DOI:** 10.1007/s00267-017-0903-7

**Published:** 2017-06-15

**Authors:** Marina Kohler, Rike Stotten, Melanie Steinbacher, Georg Leitinger, Erich Tasser, Uta Schirpke, Ulrike Tappeiner, Markus Schermer

**Affiliations:** 1 Institute of Ecology, University of Innsbruck, Sternwartestrasse 15, 6020 Innsbruck, Austria; 2 Department of Sociology, Mountain Agricultural Research Centre, University of Innsbruck, Universitätsstrasse 15, 6020 Innsbruck, Austria; 3 LEADERRegion Kufstein und Umgebung—Untere Schranne—Kaiserwinkl, Prof.-Sinwel-Weg 2, 6330 Kufstein, Austria; 4 Institute for Alpine Environment, EURAC Research, Viale Druso 1, 39100 Bozen/Bolzano, Italy

**Keywords:** Grassland farming system, European Alps, Specific resilience, Holistic scenario approach

## Abstract

Land use and land cover patterns are shaped by the interplay of human and ecological processes. Thus, heterogeneous cultural landscapes have developed, delivering multiple ecosystem services. To guarantee human well-being, the development of land use types has to be evaluated. Scenario development and land use and land cover change models are well-known tools for assessing future landscape changes. However, as social and ecological systems are inextricably linked, land use-related management decisions are difficult to identify. The concept of social-ecological resilience can thereby provide a framework for understanding complex interlinkages on multiple scales and from different disciplines. In our study site (Stubai Valley, Tyrol/Austria), we applied a sequence of steps including the characterization of the social-ecological system and identification of key drivers that influence farmers’ management decisions. We then developed three scenarios, i.e., “trend”, “positive” and “negative” future development of farming conditions and assessed respective future land use changes. Results indicate that within the “trend” and “positive” scenarios pluri-activity (various sources of income) prevents considerable changes in land use and land cover and promotes the resilience of farming systems. Contrarily, reductions in subsidies and changes in consumer behavior are the most important key drivers in the negative scenario and lead to distinct abandonment of grassland, predominantly in the sub-alpine zone of our study site. Our conceptual approach, i.e., the combination of social and ecological methods and the integration of local stakeholders’ knowledge into spatial scenario analysis, resulted in highly detailed and spatially explicit results that can provide a basis for further community development recommendations.

## Introduction

Cultural landscapes were shaped over centuries by the interplay of human activity and nature, and are thus the epitome of social-ecological systems (SES) (Farina 2000; Hanspach et al. 2014). While environmental conditions generally determine the occurrence of land cover types (e.g., forest, grassland, and aquatic systems), human activities influence land cover through specific management schemes leading to a variety of land use types (Aranzabal et al. 2008; Foley et al. 2005). Heterogeneous (multifunctional) landscapes deliver multiple ecosystem services (e.g., recreation, food production, and climate regulation) and often contain habitats with high biodiversity (Plieninger et al. 2015).

However, cultural landscapes have been undergoing significant changes in the last decades with both intensification and abandonment as opposing developments (Aranzabal et al. 2008; Plieninger et al. 2014). Various requirements such as new areas for settlement, energy production, or nature conservation, and also loss of profitability lead to the replacement of traditional land use types and sometimes to rapid and pervasive transformation of landscapes (Plieninger et al. 2014). Hence, SES are subject to complex dynamics inducing land use and land cover (LULC) change. As land use types are embedded within a social framework, management decisions are determined by factors of different disciplines with more or less strong influence on decision-making processes (Hersperger and Bürgi 2009; Kizos et al. 2014).

The concept of resilience has proven valuable in understanding the dynamics of SES (Folke 2006; Folke et al. 2010; Schermer et al. 2016). Referring to resilience here as the “capacity of a socio-ecological system to retain the same functions, structures, and identities while facing disturbances” (Walker et al. 2004), the concept serves to identify driving forces and dynamics of the social and ecological sub-systems (Folke et al. 2010; Holling 2001; Wilson 2012). Several studies focus on the drivers behind land use change and their impact on landscapes and ecosystem services (Bürgi et al. 2004; Hanspach et al. 2014; Kizos et al. 2014). The identification of these interlinkages is in turn essential to forecast future developments (Hersperger et al. 2010). Driving forces can thereby be related to changes in socio-economic, political, technological, natural, or cultural systems (Brandt et al. 1999; Bürgi et al. 2004). Multiple processes act on various temporal and spatial scales with different effect according to the scale (Plieninger et al. 2015). The processes can include different institutions or stakeholders and are often mutually dependent and difficult to identify (e.g., changes in amount of EU subsidies and changes in local consumer behavior at the same time).

Evaluating the impact of these drivers on future land use is indispensable for sustainable land management (Lindborg et al. 2009). Scenarios as a tool to demonstrate plausible futures are thereby an appropriate method for minimizing uncertainty regarding the development of cultural landscapes (Soliva et al. 2008). Plausible scenarios for SES have to be developed depending on purpose and scale (IPBES 2016). For detailed results on land use and to comprehend various dynamics within the SES, (spatially) small-scale analyses are favorable (Hanspach et al. 2014). This might be even more necessary in mountain regions because of heterogeneous terrain.

However, only few studies link the concept of social-ecological resilience to impacts on land use (e.g. van Apeldoorn et al. 2011; Colding 2007), but most analyze foremost conceptual resilience thinking (e.g., Folke et al. 2010) or model LULC changes based on digital information (e.g., Verburg et al. 2010). Here, we apply a sequence of different steps embedded in the framework of resilience to determine future land use changes: (a) analysis of the SES (farming system) characteristics by literature research and expert interviews, (b) identification of key drivers that determine farmers’ land use decisions, (c) development of three scenarios, i.e., continuation of current trend, positive interpretation of key drivers with respect to farming conditions, and negative interpretation, (d) stakeholder workshop with local farmers, (e) spatial mapping of land use change. This sequence of different steps will produce plausible mapping results within the study site (Hanspach et al. 2014; Kizos et al. 2014; Oteros-Rozas et al. 2015). As biophysical and socio-economic conditions are heterogeneous across the landscape and the impact of drivers therefore more or less influential, explicit mapping enhances accuracy of results (Bürgi et al. 2004; Hanspach et al. 2014). Here, knowledge of local farmers on site-specific conditions was transformed into spatial information as farmers mapped land use changes in grassland management according to each scenario. The approach was applied to an Alpine valley in Austria.

In the following, we give a description of our study site, position our study within the resilience framework, and present the various steps of our methodological approach. We end by discussing the plausibility of our results and the practicability of the conceptual framework.

## Materials and Methods

### Description of Study Site

**Fig. 1:**
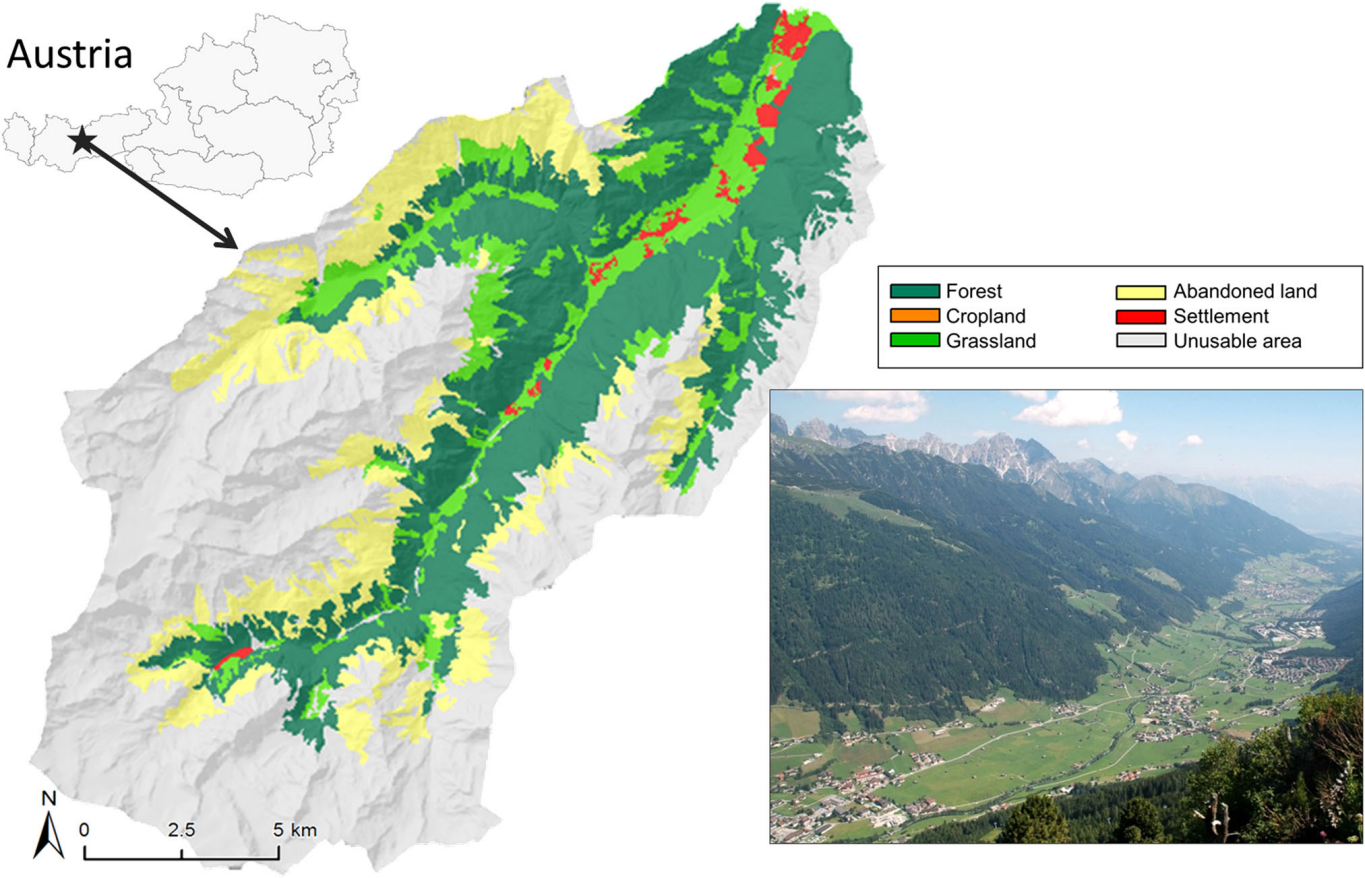
Study site “Stubai Valley” in Tyrol/Austria

Our study site (Fig. 1) comprises the municipalities of Fulpmes and Neustift in the Stubai Valley in the Central Alps (Tyrol, Austria), part of the Long-Term Socio-Ecological Research network (LTSER platform “Tyrolean Alps” site Stubai). The valley is located about 30 km south of Innsbruck, the capital of Tyrol. Around 8900 inhabitants live in Fulpmes (4250) and Neustift (4650) (Tiroler Landesregierung 2015). The study site covers around 265 km^2^ and ranges between 887 m a.s.l. on the valley floor and 3484 m a.s.l. at the highest elevation. About 73 km^2^ (27.5%) of the area is covered by forest, 23.7 km^2^ (8.9%) by managed and 40 km^2^ (15.1%) by abandoned grassland. Only 0.1% of the area is used as cropland (1% settlement, 47.4% non-usable area). A glacier offers year-round skiing facilities, which together with great scenic beauty makes the valley an attractive tourist destination in summer and winter. Tourism contributes economically to the municipalities in the valley by generating jobs as well as vitalizing the rural area in general. The proximity to Innsbruck prompts commuters to live in the valley and work in Innsbruck (Schermer et al. 2016; Statistik Austria 2015), which favours the economic situation of the Stubai Valley. A long tradition of adapted farming systems has led to the evolution of various grassland types in the Stubai Valley, providing several ecosystem services, high biodiversity, and contributing to the building of a cultural identity by inhabitants (Schirpke et al. 2013a, Soliva et al. 2008).

### Resilience Concept

**Fig. 2:**
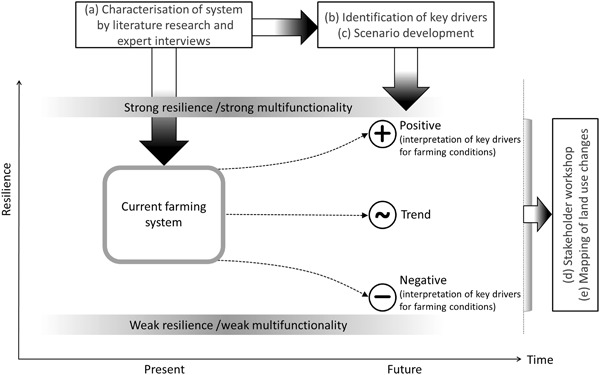
Conceptual approach embedded in the framework of resilience, after Wilson (2012)

The concept of social-ecological resilience aims to identify and evaluate insecurities that affect the current structure or processes of a system (Folke et al. 2010). By recognizing the complexities, hierarchies, and dynamics of interlinked processes, it offers a way to conceptualize uncertainty (Darnhofer 2014; Jones and Tanner 2016). In this sense, it provides a suitable framework for simplification and the identification of a reduced set of relevant interactions that influence decision processes (Holling 2001; Quinlan et al. 2016). For the purpose of evaluating resilience, many studies use surrogates such as the resilience of ecosystem service provision (Biggs et al. 2012), the transformation capacity of farms (Darnhofer et al. 2010), or the multifunctionality of rural communities (Wilson 2010, 2012). Wilson (2010) defined community resilience as the capacity of a rural community (i.e., system) to absorb shocks, and the ability to reorganize in times of change. A community can thus show strong resilience (strong multifunctionality), or weak resilience (weak multifunctionality), and thus vulnerability in its development over time (Wilson 2010, 2012). Here, we focus on a farming system on the communal level and quantify respective LULC changes in future (Fig. 2).

### Methodological Framework

The study was implemented in the Stubai Valley (Neustift) in Tyrol/Austria and consisted of the following steps:

#### Characterization of farming system

First, research reports, scientific publications, public government documents, and official agricultural statistics were analyzed to emphasize mountain grassland farming systems in general, and to identify characteristics of the local farming systems (Appendix). These data were compiled in two reports^1^: (1) The country report covers the results of a desk research on national scientific literature and includes information on the general relevance of mountain permanent grasslands, specific societal claims, formal governance mechanisms, public support systems, and suggestions for further interventions. (2) The case study report contains a description of the region, arrangement of land use types, and statistical data such as livestock units, economic situation of households as well as a description of relevant agricultural policy measures. These data were complemented by expert interviews, which gave more information about relevant actors and the current trends in agricultural development and land use at our study site.

##### Expert interviews

Semi-structured interviews with local key informants were conducted to disclose information on local farming systems and—when possible—double-checked against published data. This allowed us to discern important key drivers that influence the farming system. A key informant (expert) is considered to be a person with specific knowledge in a certain field of activity and functions as representative of a group (Bogner and Menz 2002; Flick 2007). Here, key informants had a profound knowledge of land use dynamics at the study site. They were either represented by members of the advisory service and the Chamber of Agriculture or are farmers themselves and thus have inside knowledge of farming systems and mechanisms. The sample was based on five persons: two farmers in Neustift, one of them being the head of the *Ortsbauernrat* (municipal farmers’ association); a third farmer from the nearby village of Mieders adds a reflexive perspective; the fourth farmer was the former chamber president, and the fifth key informant came from the advisory service.

The semi-structured interviews were centered around the historical development and present modes of farming systems in the valley and on the alpine pastures. Further questions were related to the factors influencing the farming practices and the future perspectives of farming in the Stubai Valley. The content-analytical evaluation of the interviews with key informants was realized according to Phillip Mayring (Mayring 2014). The text material was structured into the following inductive and deductive developed categories: labor organization, products, and farming system and land use.

#### Identification of key drivers

Key drivers that influence the system’s dynamics in Neustift have been extracted from the structured text material of the expert interviews. Single key drivers have been clustered into different categories (see Table 1). The results of all categories were again structured into the three forms of capital (Table 1) according to Wilsons’ framework of community resilience (Wilson 2010, 2012).This framework explicitly emphasizes (rural) community-environmental interlinkages, and relates key drivers of resilience to economic, social (including political and cultural) and environmental capital.

**Table 1:** Clustered key drivers identified in key informant interviews influencing grassland management decisions of farmers, grouped according to Wilson’s concept of community resilience (Wilson 2010); time horizon of scenarios ~2050

	Categories	Key drivers
Economic capital		
Village community development	Tourism	-Market for specialities, niche products, local food
		-Demand for tourist services (e.g., accommodation, living museum for tourists)
	Agricultural market	Demand for local products (e.g., Soliva et al. 2008)
	Community services	Market for community services (e.g., biogas)
	Settlement	Demand for building sites (pressure on agricultural land)
Farm management	Supplementary income	-On farm (e.g. holidays on farm, gastronomy in alpine huts)
		-Off-farm in the village (especially in tourism)
		-Off-farm (commuting to Innsbruck)
		-Time to manage grassland parallel to off-farm employment
	Farm succession	Uncertain/guaranteed
	Structural change	-Expansion or termination of business (due to market)
		-Technological *treadmill* (need for new machinery) (e.g., Schermer 2015; Schermer et al. 2016)
		-Accessibility of grassland sites and steepness (innovation produced by machinery)
Social capital		
Political intervention	Subsidies	Provision of a basic income (e.g., direct payments). In Tyrol, Austria, on average >80% of farmers’ income comes from public transfer payments (Schermer et al. 2016)
	Regulations	-Animal husbandry (e.g., barn regulation)
		-Environmental regulations (e.g., landscape conservation, protection of alpine pastures)
Farming community	Social organization (e.g., farmers, farm wives) and associations (breeders association)	-Attitude towards farming (e.g., Schermer et al. 2016)
		-Collaboration among farmers
		-Management of commons
		-Cooperation with municipality
Environmental capital		
Natural conditions—climate change	Climate change scenario based on IPCC A1B (Gobiet et al. 2014)	Need/possibilities for irrigation (increase in temperature by +1.5–2 K until 2050)
	Natural hazards	Retention sites to protect against extreme events and natural catastrophes (e.g., protective forest instead of pasture) (e.g., Beniston 2012)

#### Scenario development

Based on the identified key drivers from the expert interviews (see Table 1), three explorative scenarios (IPBES 2016) were developed. Here, scenarios are applied to examine plausible futures of complex systems under the assumption that key drivers are changing in a positive or negative way (with respect to farming conditions) and the current trend continues (Fig. 2). A comprehensive scenario includes biological, physical as well as human factors and is grounded in data, information, experiences, and estimations (Henrichs et al. 2010; van Notten et al. 2003). The storyline for each scenario was conducted by focusing on decision making processes of farmers concerning the management of grassland ecosystems (i.e., interpretation of key drivers): the trend scenario describes a possible future of the Stubai Valley following the current trend. Consequently, a positive and a negative storyline for the study region were developed to present two contrasting, but plausible futures. The time horizon is 2050 to include farm succession as a driver of land use change (several farmers will retire within the next 25 years) (e.g., MacDonald et al. 2000).

#### Stakeholder workshop

To assess the dimension of future land use change the prior developed scenarios were discussed with local stakeholders (see also Henrichs et al. 2010). First, a local expert (former researcher at the University of Innsbruck, active farmer) checked scenarios for plausibility as a pre-test before a participatory workshop was held with local Neustift farmers. The plausibility of the refined scenarios was again discussed with participants at the workshop; the scenarios correspond to the indications for participatory processes published by Henrichs et al. (2010). Farmers were invited by the head of the municipal farmers’ association (*Ortsbauernobmann*) with the aim of having a cross-section of the Neustift farming community in attendance. In total, nine farmers were present; four were over 50years, four between 40 and 50 years, and one farmer was under 35 years of age. All farmers generate additional income outside their farm, mainly within the tourism sector. The farmers’ grassland sites are dispersed throughout the Stubai Valley and cover the major part of our study site. Moreover, the farmers at least know the other sites. After presentation of the three scenarios, farmers discussed the plausibility of each one. Scenarios consequently have been adapted and mapping of land use changes was done based on the refined scenarios.

#### Mapping of land use changes

During the stakeholder workshop local farmers mapped likely land use changes according to each scenario with a pen-and-paper approach. For this purpose, the most recent orthophoto of the Stubai Valley (year 2013) was used as the basis, wherein farmers marked estimated changes with colored text markers. Farmers further added the information into which LULC type the patch will most likely transform. This process was done for all three scenarios.

The information on land use change gathered in the workshop was mapped in ArcGIS 10.1, and quantitatively analyzed. In the scenario maps, we reclassified abandoned grassland into forest when it was located below the potential treeline (~2300 m a.s.l.). Although a severe downshift in the current treeline was recorded in the Alpine region due to long-lasting anthropo-zoogenic impacts (Pecher et al. 2011), abandonment of summer pastures and the response of plants to rising temperatures will result in an upshift in the potential treeline in future (Vittoz et al. 2008).

In order to better compare LULC changes among scenarios, we used ecoregions, i.e., landscape units that share certain site characteristics such as topography, climate as well as basic socio-ecological conditions (Schirpke et al. 2012; Tasser et al. 2009). We differentiated four ecoregions: (1) valley floor (<1500 m a.s.l.), (2) forest belt, and (3) sub-alpine grassland (both between 1500 and 2300/2400 m a.s.l. with the forest belt generally at a lower elevation), and (4) alpine/nival belt (>2300/2400 m a.s.l.).

## Results

### Farming System (Step a)

In the following, characteristics of the farming system are presented.

#### Labor organization

Since the 1970s structural change has led to a decrease in employment in the agricultural sector and an increase in the tourism sector with regard to the economic situation in the Stubai Valley. This development is favoured by the valley’s environmental conditions and geographic proximity to Innsbruck (commuters). Parallel thereto, the structural change causes a shift in agricultural working structures, i.e., from main-income farming (15.5%) to part-time farming (72.6%, 2010 for Neustift, others structures are collective farms and farm associations) (Statistik Austria 2011).

However, structural change (i.e., abandonment of farms) is less pronounced in Neustift than at the state or national level in Austria. Within the last 10 years the number of farms decreased from 181 to 168, i.e., −7.2% (Statistik Austria 2011). The persistence of part-time managed farms might be attributed to pluri-active income possibilities, mainly within the tourism sector (key informants). The main income in part-time farming is obtained either on-farm (e.g., agri-tourism, direct marketing of agricultural products), or off-farm (e.g., working in the tourism sector in the valley, or commuting to Innsbruck).

#### Production system

Farming systems in the Stubai Valley can be separated into farms with animal husbandry focusing on either cattle, cows or sheep (and goats) and farms without animal husbandry, namely focusing on grassland production.

##### Cattle/cows

The main production system in the Stubai Valley is cattle breeding in combination with milk production. Main-income farmers with a focus on dairy cows keep their animals (mainly *Holstein-Fresian*) in barns in the valley (Tasser et al. 2012a). Dairy cows on alps become extinct as these cows are too heavy and their energy demand cannot be covered by mountain grasslands alone. However, suckler cows and breeding stocks are still kept on alps in summer and autumn. Common breeds are *Brown Swiss* and “Tiroler Grauvieh”, which are well adapted to mountainous terrain. Many farmers build their farming identity by breeding and are members of breeding associations (key informant).

Since 2013 dairy farmers have been delivering milk to *Milchhof Sterzing* in South Tyrol (Italy), which pays a high price for milk as compared with most other dairies in the European Union (Erker 2015). This special situation has stabilized the number of dairy farms in the Stubai Valley over the last three years (key informant). However, new EU regulations for animal husbandry (Austrian Federal Law Gazette II No. 219/2010) require barn modification with regard to free run instead of tethering. A transition period exists until 2020, but costs for a new barn run up to € 300.000 and space for modifications is limited. Consequently, it is to be expected that part-time farmers with few dairy cows and low milk production will stop farming as modifications are not profitable. As these farmers have mainly kept their cows on mountain pastures during the summer, this can result in abandonment of such pastures. Overall, the cattle stocking rate (LU ha^−1^ agricultural land) in the region is low, namely 0.6 LU ha^−1^ in 2014 (Bundesanstalt für Agrarwirtschaft 2015).

##### Sheep

Sheep and goats are often kept in combination with cattle. However, farmers with sheep only are mostly part-time farmers as this system is less labor-intensive than cattle. An increase in sheep and goat farming was recorded since 1970 (Tasser et al. 2012b), which can be due to changes in consumer demands as, for instance, an increase in allergies (cow milk). A majority of sheep farmers conduct stock breeding with a focus on meat production, whereas the breeding objective was formerly size and mobility. The dominant breed is the *Tiroler Bergschaf* (Österreichischer Bundesverband für Schafe und Ziegen 2016). It shows good surefootedness and is therefore highly suitable for grazing on unpassable grassland often above the treeline.

#### Farming systems and land use

Management schemes and land use can be differentiated according to altitude. In general, grassland steepness, proximity, and accessibility to the farm influence management intensity.

##### Valley

Grassland on the valley floor undergoes one to four cuts, and some sites are additionally grazed in spring and autumn. Meadows on hillsides are mown between one and two times (Fondevilla et al. 2016). Grassland is fertilized and produces hay or silage. Intensively used grassland is cut for the first time in mid-May, resulting in fodder with a high protein content (for dairy farming). Fertilization differs between solid and liquid manure, due to either free-run or tethering barns. With new barn requirements, the manure system shifts from solid to liquid manure. This results in larger amounts (volume) of manure, and in turn might increase the number of times the field is fertilized. In the valley there are no pastures that see year-round use. Communal areas are used as forest pastures.

##### (Sub-)alpine meadows

Today only few mown meadows exist >1600 m a.s.l. They have to be accessible and usable for machines (e.g., slope) and are mown once a year. They are always managed in combination with grazing. The majority of farmers participate in agri-environmental schemes (key informant, Tirol Atlas 2014); these are funding schemes for nature conservation measures, e.g., late mowing.

##### (Sub-)alpine pastures

Alpine pastures are stocked with either cattle or sheep, or both. Sheep pastures are mostly above 2000 m a.s.l. in the (sub-)alpine zone without consistent shepherding. Cattle pastures are usually near alpine huts and located below 2000 m a.s.l. Alpine huts with animal husbandry are generally accessible by car. They have catering facilities that provide the economic basis and main income (key informant). However, hygiene regulations cause a decline in the number of managed huts. Some (sub-)alpine pastures are collective pastures used by on average five to seven part-time farmers, as mentioned by key informants. They are extensively used, mainly grazed by sheep and young stock. Milking is still done on only a few pastures.

It can be concluded that agriculture plays only a minor direct economic role in the Stubai Valley today. Nevertheless, the existing vital farming structures and a variety of different applied management schemes contribute to vitalization of the valley. The preservation of traditional cultural landscapes remains important, especially for summer tourism.

### Key Drivers (Step b)

The identified key drivers were grouped according to Wilson (2010) and are presented in Table 1.

### Narratives of Scenarios (Step c)

Based on identified key drivers, storylines for scenarios were developed and checked for plausibility by farmers during the stakeholder workshop (step d). The most important outcomes of the plausibility discussion regarded climate change as a driver and the general influence of key drivers (see Discussion). Consequently, the negative scenario was adapted and the interpretation of key drivers relativized. The negative scenario presented here already illustrates the final adapted scenario. Overall, this resulted in more similar scenarios. The following shortened narratives of the scenarios were used in the stakeholder workshop.

#### Trend scenario

Economically, farming activities show little importance. Nevertheless, the community does value the ecological and societal services provided by the farming community. A diversified but rather extensive agriculture is mainly realized in part-time work, as tourism and commuting to Innsbruck provide good employment opportunities. Pluri-active income is important and direct payments guarantee a substantial part of it, but also demand administrative efforts. Identity building is still shaped by the local social farming institutions and basic synergies between agriculture and tourism are exploited. The continuing trend is to a slow intensification on the valley floor and a slight increase in abandonment of grassland at higher altitudes. However, even with slowly ongoing structural change the farming system is able to retain an open cultural landscape.

#### Positive scenario

The diversified agriculture within the valley efficiently cooperates with the tourism industry, e.g., providing part-time jobs, markets for local products, or clients for holidays on a farm. Still, part-time farming remains very important for supplementary farm income, but also direct payments contribute to a fixed income for farmers. Therein, self-regulating measures support collaboration among farmers. Farm succession is guaranteed and financial support/advice helps to react to new EU regulations. Even if the general development objective of the community focuses on tourism, agricultural activities are valued. Identity and overall attitude towards farming are heavily influenced by several social farming organizations in a mixture of tradition and innovation.

#### Negative scenario

Farming has little importance for food production and instead serves to sell an image for tourism purposes. Some farms generate income with holiday on farms or farm visits. Alpine pastures in certain areas function as living museums. Due to budget constraints of EU agricultural policy, direct payments do not secure an income. Many small-scale farmers have difficulties coping with new EU barn regulations and will retire. As tourism is promoted via the “new wilderness”, ecological and social services provided by farming are no longer valued by the community. Social farming organizations have lost their influence on identity building as fewer farmers continue farming.

### Land Use and Land Cover Changes (Step e)

**Fig. 3:**
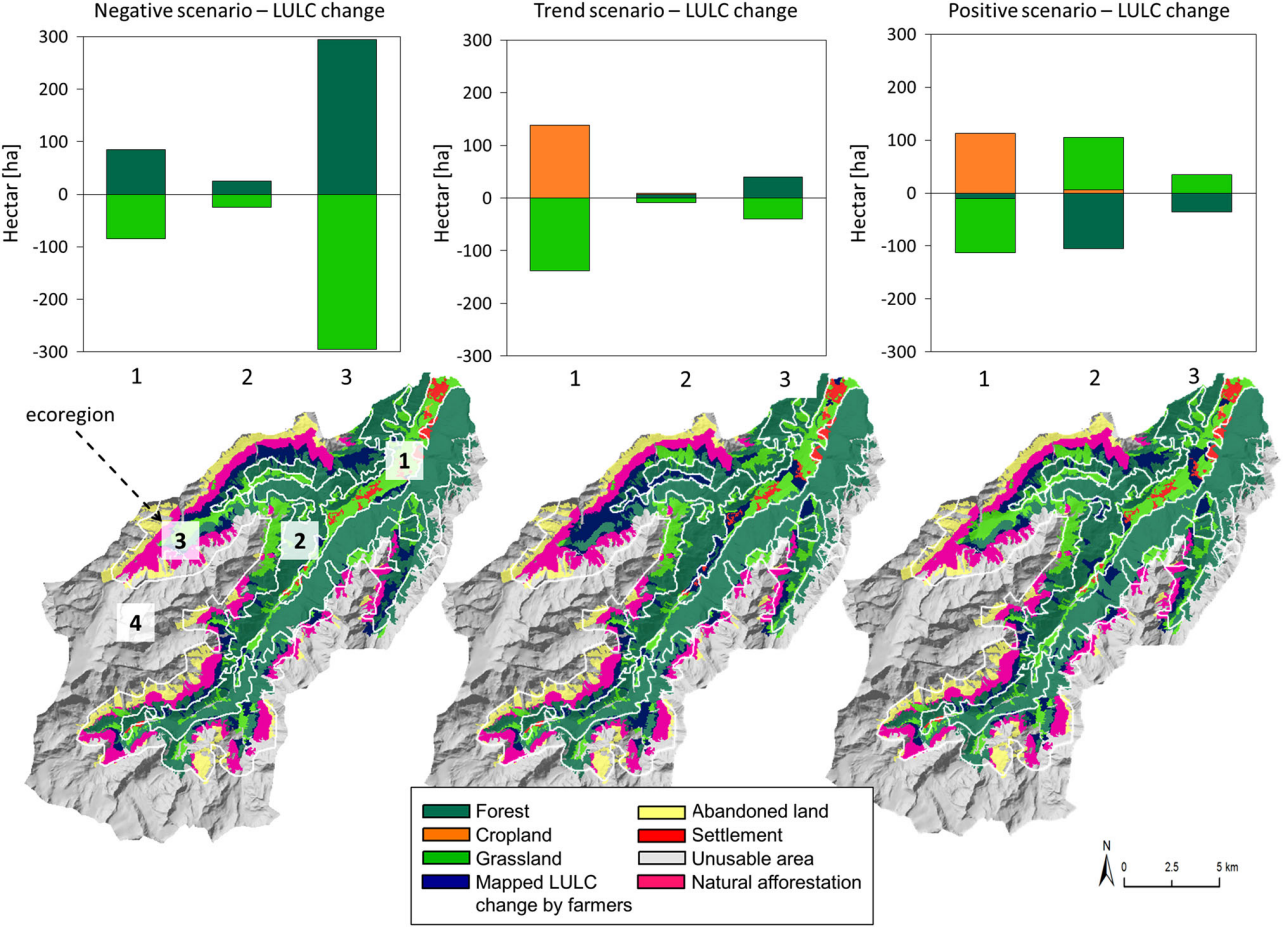
Changes in LULC (current—2050) within the ecoregions (1) valley, (2) forest belt, and (3) sub-alpine zone for the trend, positive and negative scenarios (no changes were recorded for the (4) alpine/nival belt). Within the diagrams abandonment of grassland results directly in forest (transition state “abandoned land” skipped over). Maps of the Stubai Valley show location of LULC changes mapped by farmers for each scenario. Natural afforestation (mapped in *pink*) results from grassland already abandoned today

Currently, managed grassland is mainly located on the valley floor (40%) and in the sub-alpine zone (48%), whereas 98% of the abandoned land can be found in the ecoregions at higher elevation (sub-alpine zone and alpine/nival belt). The trend scenario shows that most changes (74%) occur in the valley with a conversion of grassland into cropland; a slight abandonment of grassland and thus an increase in forest area in the sub-alpine zone (21% of changes) (Fig. 3). In the positive scenario LULC change is greatest on the valley floor (45%) and in the forest belt (42%). Similar to the trend scenario, grassland will be converted into cropland on the valley floor (Fig. 3). The conversion from forest back to grassland within ecoregions two (forest belt) and three (sub-alpine zone) in the positive scenario can be explained by reactivated forest pastures. In the negative scenario only abandonment was recorded, with highest values in the zone of sub-alpine meadows and pastures (73% of changes) (Fig. 3).

In total, LULC changes in the scenarios over all ecoregions range between 187 ha in the trend scenario and 426 ha in the negative scenario (254 ha in the positive scenario). In comparison with the total managed grassland area in our study region today (~2370.6 ha), positive and negative changes in grassland area range between 7.9% (trend) and 17.1% (negative), with the positive scenario in between (10.7%) (Fig. 3).

## Discussion

### Plausibility of Results

Agricultural intensification under the trend and positive scenarios can be linked to the predicted increase in temperature (+1.5 to 2 K), which enables cultivation of crops at the lower elevations of the valley (Gobiet et al. 2014; Schirpke et al. 2013a). However, despite higher temperatures and extreme events (climate change) causing the farmers to irrigate their sites (whenever possible), they did not intend to change their farming systems (see also Lamarque et al. 2014) by changing their management or converting grassland to cropland as they rely on fodder production for the dairy farming systems. Further, for the farmers, tourism is the most important issue. They stressed the fact that as long as tourists visit the valley, they will manage grassland sites to support a traditional and touristic picture (see also Lindemann-Matthies et al. 2010; Schirpke et al. 2013b). Grassland management on most sites will thus be continued and large-scale afforestation is not an option (as originally suggested in the negative scenario). On contrary, farmers see the possibility of recultivating forest into pastures or meadows if regulations for former managed areas are added to the currently very strict forest laws. Moreover, a favorable consumer market (demand for local products) and subsidies for extensively used pastures were assumed. In both the trend and the positive scenarios farmers emphasized the importance of having several sources of income (pluri-activity) and their self-perception as farmers (e.g., Kvakkestad et al. 2015) for the continuation of grassland management at higher elevations, including the forest belt, the sub-alpine and the alpine/nival zones (Darnhofer et al. 2010; Tasser et al. 2012a). In line with other studies (e.g., Lindborg et al. 2009), reactivated pastures and cropland were mostly located on already historically used land. The negative scenario assumed a reduction in subsidies and direct payments within the Common Agricultural Policy (Hanspach et al. 2014) as well as a trend in consumer behavior to cheaper and non-local products (Schermer et al. 2016; Soliva et al. 2008). Farmers react by ceasing to manage grassland in all ecoregions (Loibl and Walz 2010). As the management of sub-alpine meadows and pastures is favoured by subsidies, loss of these payments is seen to have its greatest influence in this zone (Lindborg et al. 2009; MacDonald et al. 2000; Schirpke et al. 2013a).

Overall, farmers do not expect distinct changes in the trend and positive scenarios. Contrasting to past large-scale abandonment or intensification processes in many alpine regions (Egarter Vigl et al. 2016), significant changes in LULC have not occurred. This can first be linked to tourist demand, proximity to Innsbruck, and thus employment possibilities outside the agricultural sector (see also Fondevilla et al. 2016; MacDonald et al. 2000). Second, historical assessment of landscape patterns in the Stubai Valley shows that significant LULC changes already took place between 1950 and 1985 (Egarter Vigl et al. 2016), and we are therefore at the end of a large-scale transformation process. However, still considerable conversion of grassland into forest (17%) was recorded for the negative scenario.

Changes in LULC induce a shift in ecosystem functions (and, after human demand, in ecosystem services) as they rely on certain ecosystem processes (e.g., nutrient cycle) provided by habitat types in varying extent (Lamarque et al. 2011). As an example, with the conversion from grassland to forest, forage quantity will decrease (or diminish) while the amount of carbon storage will increase (Burkhard et al. 2012). Therefore, impacts of LULC change must be further analyzed for their effects on multifunctional ecosystem service provision.

### Conceptual Approach

Less an explicit tool and more a guiding framework, the concept of social-ecological resilience can frame analysis of future SES development (Darnhofer 2014; van Apeldoorn et al. 2011). By recognizing the complexity of drivers and scales that influence a system’s resilience, use of the resilience concept in combination with future land use assessment proved to be successful. Application of the resilience framework contributed positively to the structuring of social, economic and environmental interlinkages and increased understanding of the system’s dynamics. We recognized the importance of focusing on a limited set of relevant indicators and thus simplifying the complexity of the system, especially in the case of our participatory approach (Helming et al. 2011). With farmers as our target group and the evaluation of impacts on respective land use management decisions, we indirectly included other scales of the SES such as e.g., institutional regulations or policy design.

Our methodological approach, namely (a) characterization of the SES (farming system) by literature research and expert interviews, (b) identification of key drivers, (c) scenario development, (d) stakeholder workshop, and (e) mapping of LULC change, resulted in a high level of detailed and spatially explicit outcomes of possible future grassland land use. By applying both scientific methods and local knowledge—first within expert interviews and second with the validation of the future scenarios by local stakeholders, we gained a deep insight into the farming system and therefore enhanced the probability of evaluating possible future LULC changes (Hanspach et al. 2014; Oteros-Rozas et al. 2015; Plieninger et al. 2013). Nevertheless, deriving from an approach of qualitative social research (expert interviews), the results of this study do not claim to be representative (see e.g., Kruse 2014). However, we claim that the same methodology could be applied in other areas to map possible futures. For its application it is always the local context and the access to the field, which are decisive for the identification of participants for the expert interviews and the stakeholder workshop; and thus, the results. However, our approach shows a high demand on time and labor and might therefore not be applicable when resources are limited, especially in regions with a great diversity in farming systems.

## Conclusion

LULC patterns are the result of social and ecological processes and one component of social-ecological resilience. Here, we used the framework of resilience to ensure that landscape scenarios contain all relevant (multi-sectoral) drivers that influence future land use. With our transdisciplinary methodological approach, we translated tacit local knowledge into spatial information, which allowed us to evaluate quantitative and spatially explicit results of grassland development. We believe that our study could serve as a basis for elaborating practical solutions and recommendations for community development or policy design to guide land use and consequently ecosystem service provision in future (Plieninger and Bieling 2013).

## Appendix

Table 2

**Table 2:** Reports analyzed for the case study “Stubai Valley”

Author/Editor, title	Source
Agrar Markt Austria (AMA), 2013. Grundsätzliches zur Vor-Ort-Kontrolle (VOK) der AMA	http://www.ama.at/Portal.Node/public?gentics.am=PCP&p.contentid=10007.28285. (Accessed 31 Oct 2013)
Agrarmarkt Austria (AMA) 2013. Maßnahmenerläuterungen ÖPUL 2007	http://www.ama.at/Portal.Node/ama/public?gentics.am=PCP&p.contentid=10007.25771 (Accessed 10 Oct 2013)
Agrarmareting Tirol (2012) Tirol Agrarmarketing—Wir über uns	http://www.amtirol.at/index.php?id=2&topId=2 (Accessed 28 Nov 2013)
Bartel A, Süßenbacher E, and Sedy K (2011) Weiterentwicklung des Agrarumweltindikators “High Nature Value Farmland” für Österreich (No. 0). Wien	
Bio AUSTRIA (2013) Grundlagen Bio AUSTRIA	
BIO AUSTRIA (2010) Produktionsrichtlinien	http://www.bio-austria.at/biobauern/richtlinien/bio_austria_richtlinien/bio_austria_produktionsrichtlinien (Accessed 28 Nov 2013)
Buchgraber K, Schaumberger A, Pötsch EM (2011) Grassland farming in Austria—status quo and future prospective. *in* Pötsch EM, Krautzer B, Hopkins A (eds) *Grassland farming and land management systems in mountainous regions*. Proceedings of the 16th Symposium of the European Grassland Federation, Gumpenstein, Austria August 29th–August 31st 2011. Grassland Science in Europe Vol. 16 (pp 13–26)	
Bundesanstalt für Alpenländische Milchwirtschaft (BAM), (2013) Über uns	http://www.bam-rotholz.at/ueber-uns/ueber-uns.html (Accessed 28 Nov 2011)
Bundesministerium für Gesundheit (2013) AGES	http://bmg.gv.at/home/Ministerium_Minister/Gesellschaften/AGES_Oesterreichische_Agentur_fuer_Gesundheit_und_Ernaehrungssicherheit (Accessed 28 Nov 2013)
European Commission (2005) Support for rural development by the European Agricultural Fund for Rural Development (EAFRD). European Council, Brussels	
European Commission (2006) Rural development policy 2007–2013—Common monitoring and evaluation framework. Guidance note B—Evaluation guidelines	http://ec.europa.eu/agriculture/rurdev/eval/index_en.htm (Accessed 28 Nov 2013)
European Commission (2012) What are EU directives	http://ec.europa.eu/eu_law/introduction/what_directive_en.htm (Accessed 28 Nov 2013)
European Evaluation Network for Rural Developments (2008) The application of the high nature value impact indicator, Brussels	http://www.efncp.org/policy/ (Accessed 10 Oct 2013)
Greif F, Parizek T, Pfusterschmid S, Wagner K (2005) Grünland in Österreich: Bewirtschaftung—Bewahrung—Förderung. Schlussbericht zum Forschungsprojekt AW/130/97„ Alpines Grünland“ der Bundesanstalt für Agrarwirtschaft	
Groier M (2007) Permanent grassland in change: aspects of grassland farming in Austria	http://www.mtnforum.org/sites/default/files/publication/files/876.pdf (Accessed 22.7.2014)
Guggenberger T, Ringdorfer F, Blaschks A, Huber R, Haselgrübler P (2014) Praxishandbuch zu Wiederbelebung von Almen mit Schafen. Lehr- und Forschungszentrum Raumberg Gumpenstein, Irdning -Donnersbachtal	
Hovorka G, Gmeiner P (2012) Die Neugestaltung der Ausgleichszulage für naturbedingte Nachteile in Österreich. *Jahrbuch der Österreichischen Gesellschaft für Agrarökonomie*, 21(2): 103–112	http://oega.boku.ac.at (Accessed 28 Nov 2013)
Hovorka G (2011) Die Evaluierung der Ausgleichszulage für naturbedingte Nachteile. Facts & Features Nr. 46 BABF, Wien	
Kirner L (2013) Prämienmodelle für die 1. Säule im Rahmen der GAP bis 2020: mögliche Auswirkungen für typische Milchviehbetriebe in Österreich. *Jahrbuch der Österreichischen*	www.boku.ac.at/oega (Accessed 28 Nov 2013)
*Gesellschaft für Agrarökonomie* 22(2): 97–106	
Land Tirol Amt der Tiroler Landesregierung Gruppe Agrar (2013) Bericht zur Lage der Tiroler Land- und Forstwirtschaft 2011/2012. Kurzbericht, Innsbruck	https://www.tirol.gv.at/landwirtschaft-forstwirtschaft/agrar/daten/gruener-bericht/. (Accessed 10 Oct 2013)
Lebensministerium (2012) Cross compliance	http://www.lebensministerium.at/en/fields/agriculture/Directpayments/Crosscompliance.html (Accessed 28 Nov 2013)
Lebensministerium (2010) Evaluierungsbericht 2010. Halbzeitbewertung des Österreichischen Programms für die Entwicklung des ländlichen Raums, Vienna	http://www.lebensministerium.at/land/laendl_entwicklung/evaluierung/le_berichte/eval.html (Accessed 28 Nov 2013)
Lebensministerium (2010) Evaluierungsbericht 2010. Teil B. Bewertung der Einzelmaßnahmen, Vienna	http://www.lebensministerium.at/land/laendl_entwicklung/evaluierung/le_berichte/eval.html (Accessed 28 Nov 2013)
Lebensministerium (2010) Evaluierungsbericht 2010. Anhang I Beauftragte Studien, Vienna	
Lebensministerium (2010) Evaluierungsbericht 2010. Teil B. Bewertung der Einzelmaßnahmen - 2. Teil, Vienna	
OECD (2013) Agricultural policy monitoring and evaluation 2013. OECD Countries and Emerging Economies OECD Publications, OECD	http://www.oecd.org/tad/agricultural-policies/monitoring-and-evaluation.htm (Accessed 28 Nov 2013)
Österreichische Bundesforste (2013) Unternehmen—Österreichische Bundesforste	http://www.bundesforste.at/index.php?id=7 (Accessed 28 Nov 2013)
Penker M (2002) Naturschutz auf landwirtschaftlichen Flächen—eine institutionenökonomische Betrachtung. *Die Bodenkultur* 53(4) pp 217–226	
Pistrich K, Wytrzens HK (2005) Leitbildanalyse und Funktionsprofil für das österreichische Grünland auf nationaler und lokaler Ebene. *Jahrbuch der Österreichischen Gesellschaft für Agrarökonomie*, Band 10, S. 107–126	www.boku.ac.at/oega
Pötsch EM (2009) Multifunktionalität und Bewirtschaftungsvielfalt im österreichischen Grünland. *Ländlicher Raum* Online-Fachzeitschrift des Bundesministeriums für Land- und Forstwirtschaft, Umwelt und Wasserwirtschaft 2009	
Statistik Austria (2013) Ein Blick auf die Gemeinde	http://www.statistik.at/blickgem/gemDetail.do?gemnr=70334 (Accessed 30 Oct 2013)
Statistik Austria (1999) Neustift Betriebserhebung	
Statistik Austria, Struktur der Betriebe. Interaktive Karte. 2013	http://www.statistik.at/web_de/interaktive_karten/064571.html (Accessed 30 Oct 2013)
Statistik Austria (2013) Tourismus in Österreich. Ergebnisse der Beherbergungsstatistik, Wien	
Suske W (2012) Evaluierung des Salzburger Regionalprojekts für Grundwasserschutz und Grünlanderhaltung	http://www.gruenerbericht.at/cm3/download/viewcategory/128-studien.html 47_salzburgerregionalprojekt_endbericht.pdf (Accessed 30 Oct 2013)
Tiroler Bauernbund (2013) Bauernband—Wir über uns	http://www.tiroler-bauernbund.at/tiroler-bauernbund/wir-ueber-uns.html (Accessed 28 Nov 2013)
Tiroler Landesregierung (2013) Planungsverband - 21 - Stubaital	http://www.tirol.gv.at/themen/zahlen-und-fakten/statistik/regionsprofile/plv21/ (Accessed 30 Oct 2013)
Zanetti P, Ornette K Sol Marte 2012, September. tirisMAPS: Protected Areas	https://portal.tirol.gv.at/mapAccelWeb/ClientServlet?CMD=Init&VIEWID=-86&MAPWIDTH=807&MAPHEIGHT=569&OVMAPWIDTH=200&OVMAPHEIGHT=122&ACTION=0&TYPE=-1&THEMEIDS=3082|3083|3660|4791|5604|2949 (Accessed 12 Sept 2012)

## Footnotes

1 For details see the country reports on the project homepage: http://www.project-regards.org/Publications.html
